# Phytocanabinoids and synthetic cannabinoids: from recreational consumption to potential therapeutic use – a review

**DOI:** 10.3389/ftox.2024.1495547

**Published:** 2025-01-28

**Authors:** Helena M. Teixeira

**Affiliations:** ^1^ Department of Research, Training and Documentation, National Institute of Legal Medicine and Forensic Sciences, Coimbra, Portugal; ^2^ Faculty of Medicine, University of Coimbra, Coimbra, Portugal

**Keywords:** phytocanabinoids, synthetic cannabinoids, recreational consumption, therapeutic use, cannabinoids

## Abstract

Cannabinoids are part of the most popular group of illicit substances in the Western world. The word “cannabinoid” refers to any chemical substance, regardless of structure or origin, that binds to the body’s cannabinoid receptors and that has effects similar to those produced by the Cannabis plant. Regarding their origin, cannabinoids can be classified into endocannabinoids, phytocannabinoids and synthetic cannabinoids. The behavioral and physiological effects of cannabinoids have received particular attention over the last few decades, including sensations of euphoria, relaxation and loss of concentration, with their repeated use being associated with short and long-term side effects, including respiratory and cardiovascular disorders, cognitive changes, psychoses, schizophrenia and mood disorders. On the other hand, recent investigations have proposed a promising therapeutic potential of cannabinoid-based drugs for a wide range of medical situations, including neurological and psychiatric disorders, among other indications. The growing popularity in the use of cannabinoid-based compounds, both for recreational and therapeutic purposes, has been accompanied by an equally continuous and growing evolution of knowledge regarding their potential harmful and beneficial effects. However, there are several open questions and challenges to be answered, which require more and better investigations. This article’s main objectives are: i) to understand the importance of the action of cannabinoids in humans; ii) identify the different types of cannabinoids that exist and understand the differences in their action; iii) distinguish the legislative framework for cannabinoid consumption; iv) identify the possible adverse effects of cannabinoid consumption, as well as their potential benefits; v) know the existing medical-scientific evidence in terms of therapeutic potential, particularly in relation to aspects of safety and efficacy; vi) encourage critical thinking about the recreational consumption and therapeutic use of cannabinoids, based both on currently available evidence and gaps in knowledge.

## 1 Introduction

According to the World Health Organization (WHO), the commonly known as “cannabis” is the most trafficked and abused illicit drug, with its consumption having an annual prevalence rate of approximately 147 million individuals, that is, almost 2. 5% of the global population (Alves et al., 2020). The Cannabis plant has perhaps been the most studied of all time in different forms, having been used for recreational, medicinal or scientific purposes due to its bioactive components (Thomas and ElSohly, 2016).

Most of the biological activity attributed to Cannabis has been associated with so-called “cannabinoids.” This term initially represented the group of typical C21 terpenophenolic compounds present in the Cannabis plant, its carboxylic acids, analogues and transformation products. However, a broader classification comprising new classes, groups and subgroups of cannabinoids has been proposed to better represent their origin and structural variety. That is, cannabinoids now constitute endogenous ligands or endocannabinoids and a whole set of herbal medicines, natural and synthetic, all active on the mentioned cannabinoid receptors. Thus, based on their origin, cannabinoids can be classified into three groups: endocannabinoids, phytocannabinoids and synthetic cannabinoids (Alves et al., 2020).

Endocannabinoids (or endogenous cannabinoids) have been identified as interfering in several physiological and pathological processes, with emphasis on the impact of the endocannabinoid system on the central nervous system (CNS), and are therefore the one that has been most intensely studied. Defined as endogenous lipids that activate specific receptors, this group of cannabinoids affects behavior in a way that recapitulates, at least partially, the effects produced by the psychoactive components of the Cannabis plant (Lu and Mackie, 2016; Hourani and Alexander, 2018).

**FIGURE 1:**
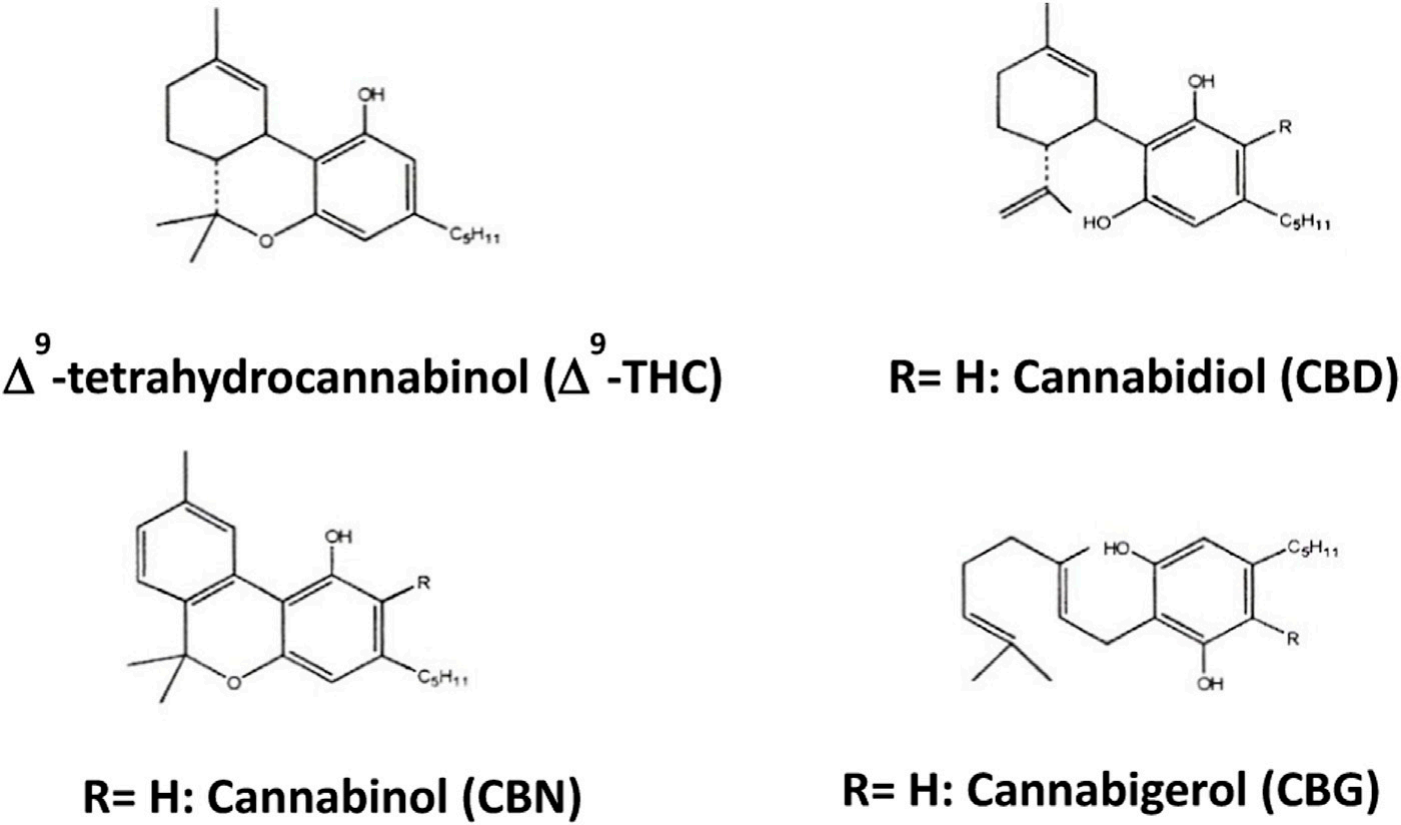
Most abundant phytocannabinoids.

Phytocannabinoids are only known to occur naturally in significant amounts in the Cannabis plant. Chemically complex, this plant contains more than 500 components, among which more than 120 cannabinoids have been isolated (Alves et al., 2020). The total number of natural compounds identified or isolated from Cannabis has continued to increase in recent decades (Bajpai and Sharma, 2016). The most abundant phytocannabinoids are Δ9-tetrahydrocannabinol (Δ9-THC), cannabinol (CBN), cannabidiol (CBD) and cannabigerol (CBG) (Figure 1).

The potential therapeutic and clinical application of phytocannabinoids has been highly appreciated in the pharmaceutical and medical areas, since their compounds have potent and different bioactivities (Andre et al., 2016; Zou & Kumar, 2018). Since the discovery of Δ9-THC, the pharmaceutical industry has carried out several studies to develop synthetic analogues, with the aim of creating compounds that express the biological activity identified in natural cannabinoids, but free from psychoactive side effects. These new molecules included not only compounds structurally similar to already known phytocannabinoids, but also compounds with a different chemical structure. Such human-synthesized, behavior-altering chemicals are called synthetic cannabinoids (SCs) (Messina et al., 2015).

Despite the growing popularity of the use of cannabinoid-based drugs, there is a lack of robust scientific studies on their toxicity and their liability for abuse, which could represent a serious threat to public health, since the risks associated with their consumption are many unexpected and unknown, thus requiring additional research in this field (Feng et al., 2017; Montesano et al., 2017; Cohen and Weinstein, 2018).

A final note highlighting the analytical challenges that clinical and forensic toxicology laboratories face when analyzing this type of substances, with the evaluation and development of analytical methodologies on alternative biological samples being crucial, as well as increasingly sensitive methods in various biological samples. On the other hand, the number of potential compounds to be investigated is large, as is the evolutionary nature of these substances, in comparison with the almost absence of analytical reference standards available (Teixeira et al., 2004; Teixeira et al. 2005; Teixeira et al., 2007; Gonçalves et al., 2019; Alves et al., 2021).

## 2 Types of cannabinoids

### 2.1 Endogenous cannabinoids or endocannabinoids

**FIGURE 2:**
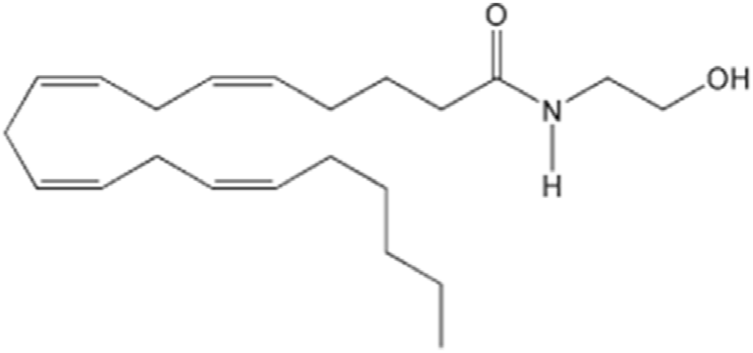
Chemical structure of anandamide.

The discovery of cannabinoid receptors sparked the need to search for the existence of an endogenous ligand with which the receptors naturally interact. The first discovered endogenous cannabinoid was arachidonylethanolamine, known as anandamide (Figure 2), which, being chemically distinct from the cannabinoids of the Cannabis plant, when compared to Δ9-THC, has a moderate affinity for CB1 receptors. However, this endogenous cannabinoid simulates the action of Δ9-THC, as it binds to both subtypes of receptors, CB1 and CB2, therefore having a similar pharmacological activity, although less potent in exerting some effects.

Anandamide has been found in several regions of the human brain (hippocampus, striatum and cerebellum) where CB1 receptors are abundant, suggesting the involvement of endogenous cannabinoids in brain functions controlled by these areas. An interesting fact is that anandamide was also found in small quantities in other regions of the body, such as the spleen, where there are high concentrations of CB2 receptors, and the heart, thus concluding that it has effects both centrally and at the peripheral level (Teixeira, 2015).

**FIGURE 3:**
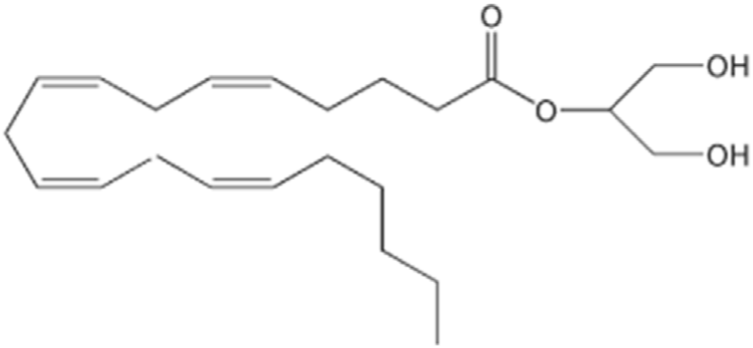
Chemical structure of 2-arachidonylglycerol (2-AG).

The effects of another endogenous compound, 2-arachidonylglycerol (2-AG) (Figure 3), were also examined through binding to a specific cannabinoid receptor, assuming that this substance may also be an endogenous ligand with a centrally relevant role. Endocannabinoids have the particular characteristic of being retrograde neurotransmitters, that is, they are synthesized in the postsynaptic neuron, exerting their function in the presynaptic one. Its synthesis is made from lipid precursors, most of which are derivatives of arachidonic acid, conjugated with ethanolamine or glycerol (Jarvis et al., 2017). Under normal conditions, the endocannabinoid system appears not to be tonically active; instead, endocannabinoids are produced according to needs, acting at a local level, being quickly inactivated by processes of cellular uptake and enzymatic hydrolysis. Significant advances in cannabinoid research have opened new frontiers, leading to an increasingly comprehensive interpretation of their effects, including the role of compounds of endogenous origin in the human body (Teixeira, 2015).

### 2.2 Phytocannabinoids

The *Cannabis sativa* plant has been cultivated for centuries due to the existence of hemp in its stems, oil in its seeds and the active biological substance (Δ9-THC) in its highest flowering parts, the chemical composition varying according to the different parts of the plant. In fact, the great interest that has been aroused for a long time is linked to the large number of chemical substances found in samples of this plant, the main class being cannabinoids. These compounds vary in number and quantity, depending on the climate, the type of soil, the variety cultivated and the way in which the cultivation was carried out. The total number of natural compounds identified or isolated from the Cannabis plant has increased in recent decades, with this plant containing more than 500 components, of which more than 120 cannabinoids have been isolated (Alves et al., 2020).

Cannabis sativa is a naturally dioecious species with male and female individuals showing unisexual flowers and characterized by sexual dimorphism: male plants are generally taller and slender than female plants and have a shorter life cycle. The chromosome set of Cannabis sativa is composed by nine pairs of autosomes and one pair of sexual chromosomes: X and Y. The male sex is endowed with an XY pair, and the female one with an XX pair. True males can be recognized by their typical morphology characterized by slender stature, few leaves and hanging inflorescences carrying male flowers and release the necessary pollen for the plant to reproduce, so it will be needed if the goal is to obtain seeds. If the goal is to maximize bud yield, it is recommended to avoid this type of plant. However, it depends on the grower and their goal whether to get rid of these plants or not. Female plants produce female pistillate flowers in dense panicles heads interspaced with leafy bracts and are the most sought after by cannabis producers since they are responsible for producing buds, the part with the highest concentration of THC (Moliterni et al., 2004; Petit et al., 2020).

Cannabis *sativa* present a large number of different chemicals, as illustrated in Table 1, being cannabinoids the main class. These compounds vary in number and quantity, according to the climate, soil type, variety cultivated and the way the crop was performed. The observed variations also depend on the part of the plant used for their extraction, the drugs preparation method for the consumption, as well as its storage conditions. *Cannabis* contains about 421 different chemical compounds, including 61 cannabinoids. During the consumption by smoking, more than 2,000 compounds can be produced by pyrolysis. Eighteen different classes of chemicals, including nitrogen compounds, amino acids, hydrocarbons, sugars and fatty acids can contribute to the single known pharmacological and toxicological properties of cannabinoids (Teixeira and Reis, 2011).

**TABLE 1:** Compounds classes found in *Cannabis sativa* (Teixeira and Reis, 2011).

Class	No. compound in the plant	Class	No. compound in the plant
Cannabinoids	61	Simple ketones	13
Cannabigerol (CBG)	6	Simple acids	20
Cannabichromene (CBC)	4	Fatty acids	12
Cannabidiol (CBD)	7	Simple esters and lactones	13
Δ^1(9)^-THC	9	Steroids	11
Δ^1(8)^-THC	2	Sugars and similar	34
Cannabiciclol (CBL)	3	Monosaccharides	13
Cannabielsoin (CBE)	3	Disaccharides	2
Cannabinol (CBN)	6	Polysaccharides	5
Cannabinodiol (CBND)	2	Cyclitols	12
Cannabitriol (CBT)	6	Amino-sugars	2
Other Cannabinoids	13	Terpenes	103
Nitrogen compounds	20	Monoterpenes	58
Quaternary bases	5	Sesquiterpenes	38
Amides	1	Diterpenes	1
Amines	12	Triterpenes	2
Alkaloids spermidines	2	Mixture of terpenoid	4
Amino Acids	18	Non-cannabinoid phenols	16
Proteins, glycoproteins & enzymes	9	Flavonoid glycosides	19
Hydrocarbons	50	Vitamins	1
Simple alcohols	7	Pigments	2
Simple aldehydes	12	Total	421

The term “cannabinoids” was attributed to the group of compounds with 21 carbon atoms present in *Cannabis sativa*, to which are added the respective carboxylic acids, analogues and possible transformation products, generally formed by three rings, cyclohexene, tetrahydropyran and benzene. It can be said that the properties of cannabinoids depend on their chemical structure, and that minimal variations in the components of the Δ9-THC molecule can cause significant changes in its activity. Of all natural cannabinoids, Δ9-THC is the compound with the greatest activity, being, together with CBN, CBD and CBG, the most abundant phytocannabinoids (Teixeira, 2015).

Δ9-THC is the cannabinoid with the greatest psychoactive potency, so this property in a Cannabis sample will depend on its content of this main compound. With regard to the other cannabinoids present in the plant and about which some information is available, CBN also has psychoactive properties, including those related to the discriminative stimuli of Δ9-THC. Specifically, compared to Δ9-THC, CBN has a higher affinity for CB2 receptors than for CB1 receptors. Given that CB2 is a peripheral receptor, it has been suggested that CBN participates in peripheral mechanisms, namely in the modulation of the immune system, an effect long attributed to cannabinoids. CBD is a cannabinoid practically devoid of psychoactive properties since it is not capable of deactivating either an agonist or an antagonist from a CB1 receptor, and as it is a non-psychoactive substance, in-depth research has been carried out to evaluate its possible effects (Teixeira, 2015). Several studies have reported that Δ9-THC is a potent activator of the CB1 receptor, while CBD does not bind directly to either the CB1 or CB2 receptors; instead, it stimulates both types of receptors. Despite this, CBD modulates the effect of Δ9-THC via direct blockade of the CB1 receptor. This modulation leads to a reduction in the unwanted side effects of Δ9-THC consumption, such as anxiety, dysphoria, panic reactions and paranoia, which is why it is also known to improve the therapeutic activity of Δ9-THC (Alves et al., 2020).

### 2.3 Synthetic cannabinoids

Synthetic cannabinoids (SCs) emerged in the 1970s, when researchers were first studying the endocannabinoid system and trying to develop new treatments for cancer pain (Lafaye et al., 2017; Papaseit et al., 2018). The first SCs were synthesized by academic laboratories and/or by the pharmaceutical industry, and the synthesis of selective cannabinoid receptor agonists with particular reference to their antinociceptive activity began at Pfizer in 1974 with CP 55,940, also known as cyclohexylphenol [2-[(1R,2R,5R)-5-Hydroxy-2-(3-hydroxypropyl)cyclohexyl]-5-(2-methyloctan-2-yl)phenol], followed by the compound HU-210, also known as 11-hydroxy-Δ8-THC-dimethylheptyl [(6aR,10aR)-9-(hydroxymethyl)-6,6-dimethyl-3-(2-methyloctan-2-yl)-6a,7,10,10a-tetrahydro-6H-benzo[c]chromen-1-ol)], synthesized in 1988 by Mechoulam’s group at the Hebrew University. John W. Huffman, Professor Emeritus of Chemistry at Clemson University in South Carolina, and his team of researchers have been involved in the synthesis of novel cannabinoids that exhibit some of the properties of Δ9-THC, and their research has focused on the synthesis of small molecules that could be applied as novel pharmaceutical analgesics, particularly molecules that bind to the CB1 and CB2 receptors. JWH-018 [Naphthalen-1-yl(1-pentyl-1H-indol-3-yl)methanone] is one of several hundred analgesic candidates that he has synthesized (Alves et al., 2020).

More than 450 SC compounds have been synthesized over 20 years, many of which bear the initials of the person/institution responsible for their synthesis, such as the following compounds ‘JWH’ by John W. Huffman, AM-2201 [1-(5-Fluoropentyl)-1H-indol-3-yl]-1-naphthalenyl-methanone by Alexandros Makriyannis, HU-210 at the Hebrew University, CP-47,497 [rel-2-((1R,3S)-3-hydroxycyclohexyl)-5-(2-methylheptan-2-yl)phenol] by Charles Pfizer or WIN 55, 212-2 [(R)-(5-methyl-3-(morpholinomethyl)-2,3-dihydro-[1,4]oxazino[2,3,4-hi]indol-6-yl)(naphthalen-1-yl)methanone]at Sterling–Winthrop, Inc., (Papaseit et al., 2018).

The continued development of synthetic variants of Δ9-THC as research tools has provided a better understanding of the physiological control system of cannabinoids in the human body, particularly in the brain, and has opened up some paths for elucidating these natural regulatory mechanisms in health and disease. That is, as these compounds have been discovered and investigated and the resulting information has been made publicly available, great advances have been made in understanding the composition and functioning of the endocannabinoid system, as well as potential therapeutic options without significant adverse effects. However, and unfortunately, several laboratories have used this knowledge for illegal purposes, including the production of illicit compounds for use as alternatives to marijuana (Zou & Kumar, 2018). Indeed, around the year 2000, SCs began to appear on the illicit drug market, with their prevalence being clearly underestimated in the “official” numbers. In 2008, forensic investigators in Germany and Austria detected the synthetic cannabinoid JWH-018 in an herbal product for the first time. Since then, its place on the black market has steadily increased (Alves et al., 2020).

Currently part of a group of substances called “new psychoactive substances” (NPS), SCs constitute the largest category in terms of the number of different substances monitored by the European Union (EU) Rapid Alert System, with a total of 190 substances notified between 2008 and 2018, and around 280 reported worldwide by the United Nations Office on Drugs and Crime (UNODOC) (Alves et al., 2020).

Commonly known as synthetic marijuana, SCs have been sold as “herbal incenses” or “herbal smoking mixtures” under different brand names. “Spice” and “K2” were the earliest in a series of SCs products sold in many European and US countries. Since then, a high number of similar products such as “Kronic,” “Cloud 9,” “Black Mamba,” “Zombie,” “Sence,” “Blue Lotus,” “Mojo,” “Moon Rocks,” “Kaos,” “Voodoo,” among others have been developed. Compared with other new drugs on the market, the increase in consumption of SCs was particularly remarkable, being its use associated with curiosity, low cost, positive drug effects including relaxation and feeling a pleasant high, belief of the products general safety, and the potential for passing drug testing (Alves et al., 2020).

## 3 Mechanism of action and pharmacodynamics

Several hypotheses have long been proposed to explain the mechanism of action of Δ9-THC, some of which have suggested that it exerts its actions through a nonspecific interaction with cell membranes and intracellular organelles. However, it is notoriously difficult to precisely delineate all the mechanisms of action of cannabinoids, given the evident activity of Δ9-THC on several targets and processes, including at the level of opiate and benzodiazepine receptors, in the synthesis of prostaglandins and even in protein metabolism. In addition, cannabinoids inhibit macromolecular metabolism, presenting marked effects at the level of enzyme systems, hormone secretion and neurotransmitters. Despite the difficulty in precisely elucidating all these effects, with the advancement of knowledge about the pharmacology of cannabinoids, it has become increasingly evident that some specific structural aspects would be necessary for cannabinoid activity, namely binding to receptors in target cells (Teixeira and Reis, 2011; Teixeira, 2015).

### 3.1 Cannabinoid receptors and mechanism of action

The action of cannabinoids through their interaction with specific endogenous receptors discovered by Devane et al. (1988) is now clearly understood, with the highest density of receptors found in basal ganglia cells, which are particularly involved in coordinating body movements. CB1 receptors mediate the majority of cannabinoid responses in the CNS, and are abundant in the cerebral cortex, hippocampus, amygdala, basal ganglia, cerebellum, thalamus and substantia nigra. The high density of these receptors in the caudate nucleus and cerebellum is consistent with the marked effects of cannabinoids on motor behaviour. Significant binding at the cerebral cortex and hippocampus correlates with effects on perception, cognition, memory, learning, endocrine function and body temperature regulation. The location of CB1 receptors in the hippocampus supports the possibility of an important role for cannabinoids in the regulation of energy and appetite. This assumption is reinforced by its presence in other peripheral organs of importance at this level, including the gastrointestinal tract, liver, skeletal muscle and adipose tissue (Teixeira, 2015).

In 1993, Munro et al. (1993) identified a second cannabinoid receptor, the CB2 receptor, which is found preferentially in cells of the immune system, outside the CNS. This distinct peripheral cannabinoid receptor appears to play an important role in immunomodulation (Lynn and Herkenham, 1994; Reggio et al., 1997), presenting relevant anti-inflammatory and immunosuppressive activity.

Ligand binding to CB1 and CB2 occurs through lateral insertion via the lipid bilayer, being the most important sequence differences between CB1 and CB2 in the N-terminal extracellular loop II (ECL2) involved in cannabinoid binding, the C-terminal sequence of TM7, and the internal C terminus itself. The other key feature in CB receptor is the presence of a toggle switch, whose activation leads to G protein binding. In CB1, the twin toggle switch involves two residues, F200 and W356 on TM3 and TM6, respectively but in CB2 it has a single toggle switch residue, W258, on TM6 and changing their relative position opens the two helices like chopsticks revealing the Gi protein binding site. Determining their status defines whether a ligand is an agonist or antagonist and these structural differences define ligand preference, with CB1 requiring the polycyclic core of the potential ligand to have a C3 alkyl chain of five or more carbons, whereas CB2 recognizes smaller classical cannabinoids (Shahbazi et al., 2020).

CB1 receptor activation has been found to increase potassium and calcium ion channel activity, modulating neurotransmitter release in a dose-dependent and pertussis toxin-sensitive manner. The receptor can exist as a homodimer, or as a heterodimer or hetero-oligomer complexed with other GPCRs and, in addition to the main binding site, the CB1 receptor also possesses an allosteric modulatory binding pocket. The CB2 receptor is closely related to the CB1 receptor, with seven transmembrane helices, a glycosylated N terminus, and the C-terminal helix embedded in the cellularmatrix (Shahbazi et al., 2020). The role of CB2 is less well defined than CB1 in the endocannabinoid system. Like CB1, CB2 also decreases the production of cAMP, although to a lesser degree, and unlike CB1, it has not been found to be coupled to G proteins other than Gi, somewhat limiting its inhibitory effect on Ca2+ and K+ channels. Also unique to the activation of CB2 receptors is an initial decrease in cAMP production, followed by a sustained increase up to 10-fold in T-cell cAMP levels, which can lead to suppression of T-cell signaling, manifesting phenotypically as an immunosuppressant effect (Bow and Rimoldi, 2016).

**FIGURE 4:**
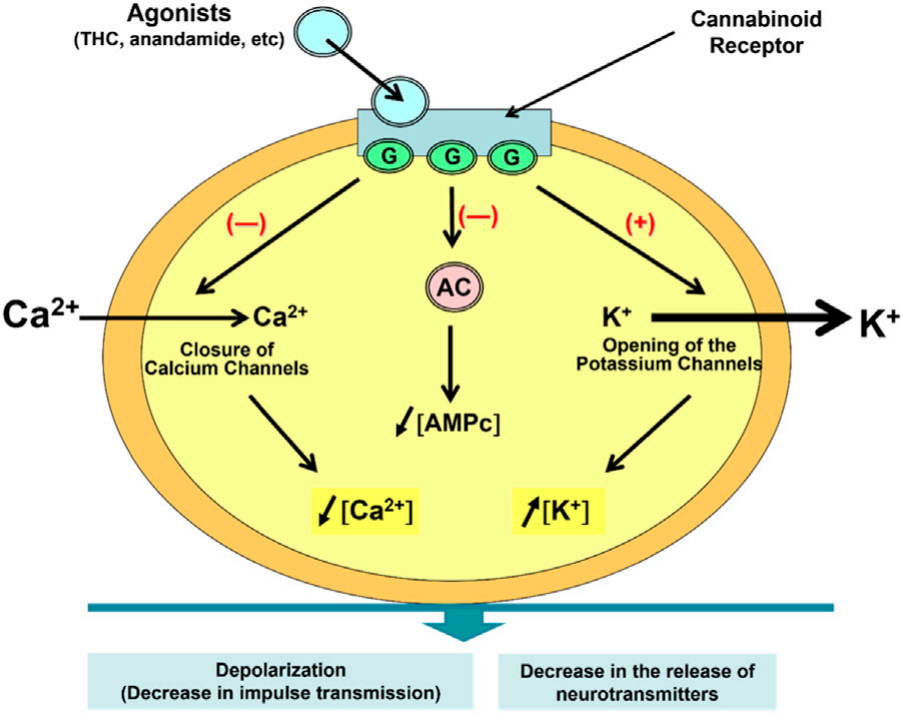
Intracellular reactions that occur when agonists interact with CB1 and CB2 receptors.

In 1986, Howlett et al. had already demonstrated that Δ9-THC inhibited the intracellular enzyme adenylate cyclase (AC) and that such inhibition only occurred in the presence of a G-protein complex, that is, in the presence of a cannabinoid receptor, which is a typical member of the largest known family of receptors: G-protein-coupled receptors, containing seven transmembrane domains (Howlett et al., 1991; Glass and Northup, 1999). The intracellular surface of receptors interacts with G proteins that regulate effector proteins such as AC, or calcium and potassium channels, and the mitogen-activated protein kinase pathway. Receptors are activated when they interact with ligands such as anandamide or Δ9-THC, and from this interaction, a series of reactions occur, including the inhibition of AC. This results in a decrease in the concentration of cAMP (Cyclic Adenosine Monophosphate) and the opening of potassium (K+) channels, decreasing signal transmission and causing the closure of calcium (Ca+2) channels, leading to presynaptic inhibition and reduced release of neurotransmitters, both excitatory (such as glutamate) and inhibitory [such as Gamma-Aminobutyric Acid (GABA)] (Figure 4) (Teixeira, 2015).

Regarding SCs, these are referred to as substances with structural characteristics that exhibit greater binding affinity to CB1 and CB2 receptors (Alves et al., 2020).

In contrast to the Cannabis plant, which mainly contains a mixture of agonist and antagonist cannabinoids, SCs compounds show differences in their selectivity, potency and function, being more potent and effective agonists of cannabinoid receptors than Δ9-THC (Cohen and Weinstein, 2018; Hourani and Alexander, 2018). Furthermore, these synthetic substances do not contain CBD-type cannabinoids that are capable of neutralizing the psychoactive properties of Δ9-THC (Altintas et al., 2016).

In general, the greater affinity of SCs for endogenous cannabinoid receptors leads to a more potent effect than natural Cannabis (Evren and Bozkurt, 2013). In particular, the JWH family of compounds, the largest, has a greater affinity for CB1 and/or CB2 receptors, being more potent than Δ9-THC, despite their chemical structures being very different from those of Δ9-THC. However, the binding affinity of SCs to the CB1 receptor can vary greatly, from being similar to Δ9-THC, as is the case with JWH-200, to being 90 times greater, as in the case of JWH-210 (Fattore and Fratta, 2011).

### 3.2 Pharmacodynamic effects

As it can be seen from their mechanism of action, cannabinoids exert multiple actions, acting on practically all biological systems. Their action is multiple and complex due to the variety of psychoactive products present in the plant, whose pharmacological and toxic actions can overlap or be additive. The main factors that influence the toxicity of these substances are: the type of cannabinoid used, the dose, the route of administration, the personality of the individual and the influence that the environment has on them, the fact that they are a novice user, the degree of habituation, their metabolism, the concomitant administration of other substances and the chronological phase of administration. Therefore, although the use of cannabinoids may have some promising effects in therapy, it is also important to evaluate their impact and adverse consequences on health (Teixeira and Castro, 2022).

Cannabinoids have multiple effects, acting on virtually all biological systems. The effects of cannabinoids have been progressively described over the last few decades, including feelings of euphoria and relaxation, changes in reaction times, lack of concentration, changes in learning and memory, or mood states (such as panic reactions and paranoia). The spectrum of behavioral effects of cannabinoids is unique, leading to the consequent classification of these substances as simultaneously stimulants, tranquilizers, or hallucinogens (Teixeira, 2015).

Other common physiological effects include increased appetite, dry mouth, vasodilation and decreased respiratory rate. Cannabinoids can affect the immune and endocrine systems and produce lung damage, with some of the effects varying considerably depending on certain aspects, including the dose (Teixeira, 2015). For example, the effect of Δ9-THC on the cardiovascular system results in a decrease in heart rate at low doses and an increase at higher doses (which can exceed 160 beats/min). Acute administration of cannabinoids in humans produces vasodilation and tachycardia, which results in a variable overall effect on systemic blood pressure. However, prolonged use of Δ9-THC results in CB1 receptor-mediated hypotension and bradycardia. Endocannabinoids induce vasodilation by acting directly on CB1 receptors located in the arterial smooth muscle of the brain. The administration of Cannabis cigarettes in normal individuals causes a slight constriction of the pupil, with vasodilation and redness of the conjunctiva being characteristic signs of Cannabis use. Other changes in vision include disturbances in color perception and adaptation to light (Teixeira, 2015).

Comparing the pharmacological similarities between SCs and Δ9-THC has been a topic of great interest among scientists and policymakers. However, little is known about the detailed pharmacology and toxicology of SCs and few well-protocoled human studies have been published. Considering the potential risks associated with SC ingestion, further pharmacokinetic and pharmacodynamic studies are needed to accurately document the consequences of consumption in clinical and forensic cases (Alves et al., 2020).

### 3.3 Recreational use and adverse effects

Cannabis is considered an illicit drug among countries that signed the 1961 United Nations Single Convention on Narcotic Drugs, but the use of cannabis or cannabis-based products as a medicine to treat defined therapeutic indications is not prevented by this Convention (EMCDDA, 2018b).

Since the 1960s, more permissive changes to laws regarding the medical and non-medical use of cannabis have been discussed, based on the perceived reduced harms of cannabis use, compared to other psychoactive drugs, and this debate has been renewed in the last decade, as some US states and Uruguay legalized the supply and use of cannabis for recreational purposes in 2012 (Silva and Carvalho, 2022).

However, proposals to legalize cannabis has raised concerns among policymakers from other countries that it may result in higher cannabis use, with an increase in its associated harms (EMCDDA, 2018b).

Since the 1990s, there has been a shift in international policies related to Cannabis and its components, moving from a more prohibitionist framework to proposals for more complex regulatory models with marked differences between countries (EMCDDA, 2018b). This is the case with some European countries, which have already chosen to introduce important regulatory changes, such as the recent regulation of the use of cannabinoids for therapeutic purposes. The debate surrounding the legalization or regulation of Cannabis is quite old, but it remains very current, both in terms of its medicinal and recreational use, with growing tolerance or even acceptance being observed, particularly in some sectors of society. At the end of 2020, and following recommendations from the WHO supported by a committee of experts, the recognition of the medicinal and therapeutic potential of cannabinoids was advocated, clearly differentiated from recreational use and whose negative implications for public health are unequivocally relevant.

According to data from the 2021 Drug Report of the European Monitoring Centre for Drugs and Drug Addiction (EMCDDA), 27.7% of the population aged 15–64 have used cannabinoids at some point in their lives. Approximately 15% of young adults aged 15–34 reported using “cannabis” in the last year, with men being twice as prevalent as women. Clearly increasing trends have been observed in most countries over the last decade, with an estimated 1.8% of adults aged 15–64 in the EU estimated, based on general population surveys, to be daily or near-daily users of “cannabis,” having used the drug on 20 or more days in the last month, and the majority (61%) being under 35 years of age (EMCDDA, 2022).

On the other hand, in contrast to the decline in the use of many NPS, such as cathinones and piperazines, the number of users of substances that mimic cannabinoids appears to be increasing. Although SCs mimic the psychotropic effects of cannabis, these compounds are recognized as being more potent than natural cannabinoids (Evren and Bozkurt, 2013; Cohen and Weinstein, 2018).

As the number of NPS detected globally has risen exponentially, the policy response of assessing and prohibiting each new substance individually has become increasingly unworkable, and in response to health-related problems associated with the consumption of SCs across Europe and the US government agencies have taken legal steps to limit the sale and distribution of these substances (Alves et al., 2020).

In Portugal, the legal regime applicable to the trafficking and consumption of narcotic drugs and psychotropic substances is approved in Decree-Law No. 15/93 of 22 January, an integral part of the Penal Code and commonly known as the “Drugs Law.” With the introduction of Law No. 30/2000 of 29 November 2000, the consumption of narcotics was decriminalised through an administrative prohibition, i.e. replacing penalties with sanctions of mere social order. The principles underlying this legal regime are linked to a different conception of the phenomenon of drug addiction, which is in line with a greater recognition of human dignity, and now sees the person with drug addiction not as a criminal but as a patient. Hence the consequent responsibility of the State in terms of realising the constitutional right to health. In short, it is thus demonstrated that, in Portugal, trafficking is prohibited and consumption is decriminalised, although not liberalised. However, there is very important legislation from a forensic point of view which clearly shows that consumption can lead to offences, and its association with road driving is reprehensible and punishable by law.

This entire legislative context has put the growing trend of cannabinoid consumption to the test, stimulating the search for compounds that provide the same effects or even enhance them, without their consumption or possession constituting an illegality. This has led to the production of a varied number of NPS, including SCs, normally included in commercial products, sold in various forms over the internet or in establishments commonly known as “smartshops” or “head shops”. The marketing of this type of substances in these formats is accompanied by “advertising” that they are legal drugs, since these substances were not included in the tables that determine prohibition.

In this context, and given the complexity and highly dynamic nature of the NPS market, Portugal has adopted specific legislation to curb the rapid proliferation of these substances. The Autonomous Region of Madeira was the first region in the country to take specific measures, through the implementation of Regional Legislative Decree No. 28/2012/M of 25 October 2012, which prohibited the sale and distribution of these substances. The following year, Decree-Law No. 54/2013 of 17 April was introduced in Portugal, which considers “new psychoactive substances” to be substances not specifically classified and controlled under specific legislation which, in their pure state or in a preparation, may pose a threat to public health comparable to that of the substances covered by Decree-Law No. 15/93, with a danger to life or to health and physical integrity. This decree therefore prohibits the production, import, export, advertising, distribution, sale, possession or provision of NSP, except when intended for industrial purposes or pharmaceutical use, provided that they are duly authorized by INFARMED (National Authority for Medicines and Health Products, I.P).

Some of the effects of cannabinoids on cognitive level have been previously described, such as euphoria, changes in reaction times, lack of concentration, changes in learning, memory or mood (Teixeira, 2015), as well as some capacities of cannabinoids to produce rapid changes on a physiological level, namely an increase in heart rate and diastolic blood pressure, vasodilation and a decrease in respiratory rate and, finally, it has been reported that cannabinoids can affect the immune and endocrine systems, cause lung damage and influence neonatal and child development (Benowitz et al., 1979; Law et al., 1984; Hollister, 1986; Day et al., 1991; Tashkin et al., 1991; Chandler et al., 1996; Fried et al., 1999; Fried and Smith, 2001).

SCs can also produce a wide range of physiological and psychiatric adverse effects, which vary in duration and severity, due to the constant changes in the composition of these synthetic substances by producers in order to avoid detection and regulation. These mitigation strategies make the treatment of their toxicity particularly challenging, as the compounds vary greatly in potency, efficacy and duration of action, making their effects unpredictable, resulting in different experiences for consumers (Brents and Prather, 2014).

Similar to phytocannabinoids, the psychoactive effects of SCs range from a pleasant and desirable euphoria to states of anxiety, relaxation, agitation and changes in cognitive abilities, including alteration of perception, time and space, in addition to possible mild cognitive impairments (Cohen and Weinstein, 2018). Serious effects observed include seizures, cardiovascular and renal toxicity, stroke, psychosis, paranoia, aggression, anxiety attacks, dependence or even death (by suicide, adverse reaction or overdose) (Evren and Bozkurt, 2013; Tournebize et al., 2017). According to several case reports, the use of SCs may also be associated with an increased risk of suicidal ideation (Tournebize et al., 2017; Cohen and Weinstein, 2018).

Several studies demonstrate an association between repeated use of cannabinoids and long-term cognitive impairment, as well as an increased risk of developing a variety of mental disorders, including bipolar disorder, depression, and schizophrenia. There is growing evidence that SCs are associated with severe deleterious psychiatric conditions. Indeed, repeated exposures to these synthetic drugs induce general negative side effects that are more severe and long-lasting than those associated with Δ9-THC (Cohen and Weinstein, 2018). Adverse effects of intoxication have been reported to occur even in those who only used SCs once, whereas withdrawal from SCs has been reported to occur only in daily users (Alves et al., 2020). Table 2 seeks to summarize the effects caused by phytocannabinoids and synthetic cannabinoids.

**TABLE 2:** Effects caused by phytocannabinoids and synthetic cannabinoids.(Adapted from Cohen and Weinstein, 2018).

Symptoms	Type of effect	Type of Cannabinoid	
		Phytocannabinoids	Synthetic cannabinoids
Neuropsychiatric	Acute	Hallucinations, altered perception, paranoia, aggression and psychosis	Hallucinations, altered perception, paranoia, aggression, psychosis and auditory and visual disturbances
	Long-term	Increased risk of psychotic disorders	Increased risk of psychotic disorders
Affect	Acute	Ansiedade e ataques de pânico	Ataques de pânico, comportamento maníaco, depressão e ideação suicida
	Long-term	Increased risk of anxiety and mood disorders	Depression, irritability and persistent anxiety
Cognitive	Acute	Attention deficit, memory, cognitive and psychomotor inhibition	Severe cognitive impairments: memory impairment, difficulty paying attention and amnesia
	Long-term	Défice aprendizagem verbal, atenção, memória e funções psicomotoras	Deficit in verbal learning, attention, memory and psychomotor functions
Cardiovascular	Acute	↑ Cardiovascular activity: ↑ heart rate and ↓ blood pressure	Tachycardia, hypertension, myocardial infarction, arrhythmias, chest pain and palpitations
	Long-term	Increased risk of cardiovascular disease	Increased risk of cardiovascular disease
Neurologic	Acute	Dizziness, drowsiness and muscle tension	Dizziness, drowsiness, convulsions, changes in sensitivity and fasciculations
	Long-term	Structural and functional abnormalities in various areas of the brain	Preliminary evidence for structural and functional alterations of the CNS
Gastrointestinal	Acute	Nausea, vomiting and change in appetite	Nausea, vomiting and change in appetite
	Long-term	Diminuição do peso	Diminuição severa do peso
Other	Acute	Bronchodilation; Influence on road driving	Acute kidney injury, abdominal pain, dry mouth, hyperthermia, fatigue, cough, influence on road driving
	Long-term	Increased risk of lung, mouth, pharynx and esophageal cancer; risk of dependence, tolerance and withdrawal	Kidney disease, insomnia, nightmares, dependence, tolerance and withdrawal

### 3.4 Therapeutic use and beneficial effects

An important distinction must be made between the different forms of Cannabis and cannabinoid preparations for medical use, differentiating between those that have obtained a marketing authorisation for medical use and those that have not. Such authorisation means that an application for a medicinal product has been submitted to a health authority which, after due evaluation, has approved it. This generally implies that the product has been investigated in extensive clinical trials and that its safety, efficacy and potential adverse effects have been assessed, as well as whether it has been manufactured to the required level of quality (EMCDDA, 2018a).

In Portugal, in 2018, the Assembly of the Republic approved Law No. 33/2018, which regulates, for the first time, the use of medicines, preparations and substances based on the Cannabis plant for medicinal purposes, which must be done exclusively with a special medical prescription, with INFARMED being responsible for regulating and supervising all activities related to the medicinal use of the plant (INFARMED, 2019). Within this regulatory framework, the principles and objectives regarding prescription, dispensing in pharmacies, possession and transportation, scientific research, regulation and supervision of activities related to the use of the Cannabis plant for medicinal purposes and information to professionals were established, which were regulated by Decree-Law No. 8/2019, of January 15.

In 2019, INFARMED published resolution no. 11/CD/2019, which describes the therapeutic indications considered appropriate for preparations and substances based on the Cannabis plant (INFARMED, 2019). The published document states that, given their characteristics, the prescription of these products is limited to the following situations: i) preparations and substances that have a marketing authorisation granted by INFARMED; ii) cases in which conventional treatments have not produced the expected effects or have caused relevant adverse effects; iii) the therapeutic indications listed in the aforementioned resolution.

Finally, it should be noted that the introduction into the market of medicinal products based on the Cannabis plant for medicinal purposes is subject to a different marketing authorisation, in accordance with the rules of the Statute of the Medicinal Product. In the case of preparations/substances based on the Cannabis plant for medicinal purposes, it is necessary to obtain a distinct authorisation, in accordance with the rules of Decree-Law No. 8/2019, of January 15.

As mentioned above, in recent decades, scientific and clinical interest in the beneficial effects of cannabinoid use with therapeutic potential has increased significantly. Despite the known acute and chronic side effects of cannabinoid use, there is a growing body of scientific evidence demonstrating an equally broad therapeutic potential. This is probably facilitated, in part, by the fact that certain cannabinoids, such as CBD, have been well studied and have demonstrated a good tolerance profile and even safety in humans, even at high doses and chronically (Sachs et al., 2015).

However, cannabinoid preparations can have a highly variable composition, depending on, for example, the plant variety, the growing conditions and how the preparations are stored. This means that it can be difficult to assess their efficacy in clinical trials. Despite this, the data already available from controlled clinical trials indicate that cannabinoids alleviate the symptoms of some diseases. Hence, they are often used as an adjunctive intervention, i.e. added to other medical treatments, but not used alone. Furthermore, they are generally only used when a patient has not responded effectively to the treatments usually recommended for these disorders (EMCDDA, 2018a).

Some of these potential clinical situations, those that have been most studied and described in the literature, will be briefly discussed below:

#### 3.4.1 For neuropathic pain and spasticity in multiple sclerosis

Multiple sclerosis (MS) is a potentially disabling auto-immune disorder of the central nervous system (CNS), in which the immune system causes the destruction of myelin, the protective sheath of nerve fibers in the brain and spinal cord, and among the several symptoms associated with MS, spasticity is one of the most frequent. Presently, there is no cure for MS or MS-associated spasticity, although current management therapies may help slow down the progression of the disease (Silva and Carvalho, 2022).

In fact, due to their ability to reduce glutamate excitotoxicity, exogenous cannabinoids arise as an alternative to reduce nerve loss in MS patients (Silva and Carvalho, 2022).

Clinical trials have evaluated the efficacy of cannabinoids in the treatment of muscle spasms and neuropathic pain in patients with MS, demonstrating evidence of therapeutic effects on some of the symptoms associated with neurological disorders, chronic pain, and other conditions for which cannabinoids appear to provide relief (Sachs et al., 2015; Cohen et al., 2019).

The most widely tested product has been nabiximol (Sativex), a standardized cannabis extract with approximately equal amounts of Δ9-THC and CBD, administered as an oral spray, but there is moderate evidence that nabiximols may be used in the treatment of MS-related spasticity. For example, small improvements in spasticity were observed in MS patients given nabiximols, compared to placebo (total of six randomized clinical trials), although no statistical significance was observed in most studies Other studies have reported improvements in symptoms related to spasticity, like incontinence, or pain, rather than spasticity itself (Silva and Carvalho, 2022).

The first randomized study carried out with Sativex^®^ took place in 2007 and included a total of 189 patients with spasticity due to multiple sclerosis. The study was double-blind and evaluated a group that was treated with Sativex^®^ daily and another that received a placebo, over a period of 6 weeks. Sativex^®^ demonstrated that the active ingredient had better subjective (the patient quantified the spasms he had) and objective (using the Ashworth scale) efficacy than placebo, with statistical significance and the authors reported that around 40% of patients had a favourable response (Collin et al., 2007). In 2010, the same main author published another study with 337 patients with refractory spasticity due to multiple sclerosis over a period of 15 weeks. This consisted of a double-blind and randomized intention-to-treat analysis. The authors concluded that Sativex^®^ led to a significant reduction in symptoms and that the response obtained in the first 4 weeks of treatment seemed to be a useful predictor for the effectiveness of the therapy (Collin et al., 2010).

In response to previous findings, Novotna et al. (2011) conducted a double-blind, randomized trial involving 572 patients with spasticity resistant to standard therapy over 19 weeks. Patients underwent single-blind adjuvant therapy with Sativex^®^ for 4 weeks and those who achieved a clinical improvement equal to or greater than 20% entered the next phase: a randomized placebo-controlled follow-up for 12 weeks. The authors defended this methodology as they considered it reflected clinical practice. Of the initial sample of 572 patients, 272 improved and progressed to the 12-week study. From here, 241 were randomized. The intention-to-treat analysis showed a significant difference in favour of the active ingredient, as other parameters also showed significant improvement (subjective and objective analysis of the patient, quality of sleep, global impression of the caregiver) (Novotna et al., 2011).

In randomized clinical trials, some patients who received nabiximol (in addition to existing treatment) reported less muscle spasticity than those who received placebo. However, physician assessments of patients’ muscle spasticity showed only marginal reductions, with some investigators describing the efficacy data as being of “moderate” quality (EMCDDA, 2018a).

Additionally, it is important to note that although cannabis-based medications have improved certain MS symptoms, some studies have shown adverse effects on cognitive and physical function that accompany the relief of these MS-related symptoms (Ghaffar and Feinstein, 2008; Honarmand et al., 2011; Pavisian et al., 2014).

In this sense, it seems reasonable that MS patients are primarily treated with conventional therapies and that nabiximols may only be used in cases that do not respond to such standard therapies.

Parkinson’s disease is a neurodegenerative condition associated with the loss of dopaminergic neurons in the basal ganglia, involved in motor coordination. In addition to dopaminergic receptors, these neurons also have cannabinoid receptors (Fernández-Ruiz et al., 2015). It is known that the activation of CB1 receptors can stimulate the dopaminergic system, justifying the theoretical potential of medicinal cannabis in this context (Gilgun-Sherki et al., 2003). Furthermore, it has been described that Parkinson’s disease is associated with a reduction in the expression of CB1 receptors in brain regions associated with motor skills (Richfield and Herkenham, 1994). García et al. (2015) even reported that degenerated neurons in the substantia nigra expressed CB2 receptors.

There are, at least, three controlled studies involving the use of medicinal cannabis in Parkinson’s patients, with some authors studying 21 patients and reporting an improvement in quality of life with oral CBD, but without significant effects on motor functioning or neuroprotection (Grotenhermen and Müller-Vahl, 2016).

Huntington’s disease is a rarer entity and is characterized by changes in movement (chorea) and behaviour, often culminating in dementia. The endocannabinoid system may be involved in the disease, but there is no convincing evidence of the effectiveness of cannabis in this context. Palazuelos et al. (2009) reported that CB2 receptors expressed on microglial cells may have a protective role in Huntington’s disease (Palazuelos et al., 2009). Regarding Alzheimer’s disease, the effects of CB1 receptors are not clear: there are reports that their expression is increased in asymptomatic patients at an early stage of the disease; on the other hand, there are reports of widespread loss of them in the plate areas (Farkas et al., 2012; Ramírez et al., 2005).

In summary, the endocannabinoid system may be related to neurodegenerative diseases, but there is no evidence to date that justifies the use of cannabis in this context.

#### 3.4.2 For chronic non-cancer pain

One of the most commonly cited reasons why patients use cannabis for medical purposes in the United States is to treat chronic non-cancer pain, including neuropathic pain, arthritis, back pain, neck pain, shoulder pain and headaches (EMCDDA, 2018a).

Cannabinoids have been suggested to have an antinociceptive action mostly through the activation of TRPV1 or the metabotropic GPR18 and GPR55 receptors (Silva and Carvalho, 2022).

There are several controlled studies that have investigated the effects in treating neuropathic and chronic pain in humans. Most were performed with products such as nabilone, dronabinol and Sativex^®^/Nabiximols^®^. These compounds have been studied in both chronic and neuropathic pain and, overall, appear to have a positive effect. Additionally, the effectiveness appears to apply to all types of pain. Sativex^®^/Nabiximols^®^ is one of the few formulations currently licensed for pain control in several countries. Therefore, its effectiveness is supported by more substantial evidence. One of the pioneering studies was conducted by Berman et al. (2004), involving patients with brachial plexus injury (this type of injury is considered a good model of central neuropathic pain). 48 patients with at least one injured nerve root were involved and the double-blind study was carried out in a randomized manner. A placebo comparison was made with two cannabis formulations–Nabiximols^®^ and a compound containing mainly THC. The primary outcome of decreasing pain severity by two points was not met, but quality of life would have been higher with cannabinoid products due to better sleep quality Berman et al. (2004).

Regarding Sativex^®^, it demonstrated significant potential in relieving pain and improving sleep quality in patients with peripheral neuropathic pain. A clinical trial carried out at the University of California demonstrated that vaporized cannabis was effective in reducing peripheral and central neuropathic pain when compared to placebo, using both low (1.29%) and moderate (3.5%) doses of THC. In this trial, patients had acquired resistance to conventional therapy. The authors further report that the neurocognitive effects were minimal (Grotenhermen and Müller-Vahl, 2016).

Turcotte et al. (2015) conducted a randomized, double-blind study with 15 patients with neuropathic pain in the context of multiple sclerosis. The study compared nabilone with gabapentin. The selected patients were taking gabapentin without effective pain control. Therefore, they received adjunctive therapy, either with nabilone or placebo, for 9 weeks. Nabilone was shown to be effective as adjunctive therapy to gabapentin and the adverse effects were well tolerated (Turcotte et al., 2015).

Dronabinol also appears to be effective as an adjunctive therapy for chronic pain. Narang et al. (2008) studied 30 patients taking opioids for chronic pain. Phase 1 of the study consisted of a double-blind study, in which patients were randomly allocated to take 10 mg vs. 20 mg dronabinol vs. placebo. Patients administered dronabinol experienced reduced pain intensity and greater satisfaction compared to placebo, with no significant difference between doses. Phase 2 consisted of a titration of dronabinol as adjuvant therapy to opioids. There was evidence of pain relief here, compared to just standard therapy. Adverse effects were relatively mild and dependent on the dose administered (Narang et al., 2008).

More recently, Stockings et al. (2018a) reviewed controlled trials and observational studies in order to compare the effectiveness of cannabinoids versus placebo in treating various types of chronic non-cancer pain. 91 publications were collected, including 47 randomized controlled trials and 57 observational studies, totaling 9,958 participants. Patients with chronic non-cancer pain who experienced a significant reduction in pain intensity was, on average, 29% in those treated with cannabinoids and 26% in those treated with placebo. However, the proportion of patients reporting adverse effects was higher in the group treated with cannabinoids (Stockings et al., 2018b).

It is concluded, therefore, that the evidence of effectiveness in the treatment of chronic pain still leaves a lot to be desired. The challenge of developing cannabis-based analgesia with compounds that have therapeutic efficacy without undesirable neuro-behavioral effects remains.

In fact, Cannabinoids are particularly used to treat chronic neuropathic pain, especially when conventional methods have not proven effective. However, some clinical trials have shown inconclusive results, reporting adverse effects that would prohibit its therapeutic use, including drowsiness, dizziness, ataxia and blurred vision. Alarming adverse reactions have been observed particularly at higher doses (Cohen et al., 2019).

#### 3.4.3 As antiemetics

Chemotherapeutic drugs may cause nausea and vomiting as a result of their activation of neurotransmitter receptors present in the brain’s area postrema or in the terminal ends of the vagal afferents near the enterochromaffin cells in the intestine. These afferent fibers send the stimuli to the brainstem, which then processes the emetic reflex and triggers efferent signals to other organs that stimulate vomiting. Cannabinoids may display an anti-emetic activity by activating CB1 and 5-hydroxytryptamine 3 (5-HT3) receptors in the dorsal vagal complex (DVC), which regulates emesis, especially the area postrema. Specifically, studies in animal models have shown that cannabinoids may control emesis, either allosterically inhibiting 5-HT3 receptors in the DVC, or by activating presynaptic CB1 receptors, which subsequently results in a decrease of serotonin release into the synapse (Silva and Carvalho, 2022).

A systematic review, with 30 randomized comparison studies between cannabis associated with placebo vs. an antiemetic, involved a total of 1,366 patients. The oral cannabinoids nabilone, dronabinol and levonantradol produced a significantly greater antiemetic effect, compared to several drugs such as metoclopramide, chlorpromazine, haloperidol or alizapride (Abrams and Guzman, 2015). In a clinical trial, involving 61 patients, it was demonstrated that THC was as effective as ondansetron, the current drug of choice for the treatment of nausea and vomiting after chemotherapy. It should also be added that the intensity of nausea and vomiting was lower in patients using THC. Another study, which evaluated the same problem, creating a sample of two patients unresponsive to standard therapy, with seven patients receiving Sativex^®^ cannabis extract and nine receiving only placebo, concluded that a higher percentage of patients experienced a complete remission of adverse effects during a period of 5 days post-chemotherapy, when compared to placebo (22.2%) (Grotenhermen and Müller-Vahl, 2016).

An analysis of 28 clinical trials assessing the efficacy of cannabinoids to treat nausea and vomiting due to chemotherapy showed no statistically significant effect of cannabinoids compared to active comparators (e.g., prochlorperazine, chlorpromazine, domperidone) or placebo. However, the average number of patients showing complete nausea and vomiting response was higher in those individuals treated with cannabinoids (dronabinol or nabiximols) compared to placebo. In patients with the human immunodeficiency virus (HIV), cannabinoids were shown to increase weight and appetite but failed to reduce nausea and vomiting. On the other hand, the use of cannabis-based products in the treatment of nausea and vomiting resulting from medication for hepatitis C is scarce and has not shown any statistical significance (Silva and Carvalho, 2022).

In fact, the antiemetic effects of Δ9-THC (administered orally) have been compared in controlled clinical trials with those produced by placebo or another antiemetic drug in patients with nausea and vomiting related to cancer chemotherapy treatment. The results showed that Δ9-THC and other cannabinoids that produce similar effects (known as cannabinoid agonists) were more effective than placebo and often had similar levels of efficacy to the antiemetics with which they were compared. However, it is important to note that many of the clinical trials have important limitations, as newer chemotherapy regimens produce less nausea and vomiting than treatments used in trials conducted in the 1970s and 1990s, and there are still very few clinical trials comparing the use of cannabinoids with newer drugs and/or more current chemotherapy regimens (EMCDDA, 2018a).

#### 3.4.4 For palliative care in oncology

The therapeutic use of cannabis and cannabinoids has been advocated to control a wide range of symptoms reported by patients with terminal cancer, i.e., through pain control, appetite stimulation, anxiety reduction and sleep improvement.

For many centuries, the appetite-inducing effect of cannabis has been documented. Foltin et al. (1988) reported that inhalation or per os consumption of THC correlated with increased food consumption, increased caloric intake, and weight (Foltin et al., 1988). Theoretically, this effect could be explained by the high concentration of CB1 receptors in the brain’s satiety centers. The endocannabinoid system will probably also play a role in lipid and glucose metabolism (Farrimond et al., 2011).

Appetite stimulation and weight gain, both in AIDS patients and neoplastic patients, have been the subject of several studies. The effects of THC on taste, olfactory perception, appetite, caloric intake and quality of life were investigated by Brisbois et al. (2011), in adults with advanced stage cancer and compromised appetite. The 46 patients in the study were administered either 2.5 mg THC twice daily or identical placebo capsules over a period of 18 days. Patients treated with THC significantly reported that the food was tastier. Appetite before meals and calories consumed as protein increased significantly compared to placebo. Additionally, patients treated with THC reported greater quality of sleep and relaxation (Brisbois et al., 2011).

Beal et al. (1995) conducted a double-blind randomized trial on 139 patients with AIDS-induced anorexia. Patients were treated with either 2.5 mg of dronabinol or placebo. It was found that dronabinol significantly increased appetite, showing a tendency to improve body weight and mood (Beal et al., 1995). They also carried out a 12-month follow-up of patients, demonstrating that dronabinol was safe and continued to be effective (Beal et al., 1997).

However, there are reports that dronabinol is not as effective as megestrol acetate (an appetite stimulant). In fact, Jatoi et al. (2002) reported that dronabinol was less effective in improving appetite and weight in cancer patients, and Timpone et al. (1997) reported that dronabinol was only effective in combination with megesterol in patients with HIV (Jatoi et al., 2002; Timpone et al., 1997).

Mücke et al. (2018) conducted a systematic review and meta-analysis of the efficacy, tolerability and safety of cannabinoids in palliative medicine, finding no significant differences between cannabinoids and placebo in improving energy, appetite, nausea or vomiting, pain or sleep in patients with terminal cancer, nor did they find high significant evidence that cannabinoids were useful in the treatment of anorexia or cachexia. The robustness of these conclusions is limited by the small number of high-quality studies and small sample sizes, which reduce the possibility of stating differences in favour of cannabinoids. Therefore, it is important that more trials are carried out, with a larger number of individuals and better designed, to better assess the usefulness of Cannabis and cannabinoids in palliative care in oncology (Mücke et al., 2018).

#### 3.4.5 As antiepileptics

CBD has been reported to exert an overall inhibitory effect on sodium and calcium channels, which modulates the membrane electrical potential and subsequently reduces neuronal hyperactivity, suggesting its potential use in the treatment of epilepsy. Such an effect may be achieved through the desensitization of the TRPV1 channels, or by acting as an antagonist at GPR55 receptors (Silva and Carvalho, 2022).

Studies in animal models have demonstrated the antiepileptic potential of cannabinoids, suggesting that CBD may increase efficacy in preclinical models of epilepsy (McPartland et al., 2015). However, controlled clinical trials are still scarce. Other authors have concluded that short-term daily use of cannabis is safe in individuals with epilepsy; however, there is not yet sufficient evidence to draw a robust conclusion about efficacy (Gloss and Vickrey, 2014; Volkow et al., 2014).

Some studies have shown promising effects in the treatment of children with epilepsy resistant to classical treatment, with evidence of efficacy of CBD in reducing the frequency of seizures (Huntsman et al., 2020). Other studies, despite having demonstrated that the addition of CBD to conventional antiepileptic drugs significantly reduced the frequency of seizures in children with Dravet syndrome or Lennox-Gastaut syndrome, concluded that better designed and controlled clinical trials are needed to determine the doses of CBD that reliably produce antiepileptic effects, with no significant adverse events and minimal interaction with other antiepileptics (Stockings et al., 2018b).

#### 3.4.6 Other medical uses of cannabinoids

Patient groups and some physicians have advocated the use of cannabis and cannabinoids to treat a variety of conditions beyond those described above. These include a variety of disorders, such as anxiety, post-traumatic stress disorder, depression, and sleep disorders, among others.

However, research on the therapeutic benefits of cannabinoids in these psychological conditions is also scarce, with some evidence that they may have a beneficial impact on sleep quality in individuals with post-traumatic stress disorder. However, further studies are needed to obtain more robust evidence of the therapeutic effects of cannabinoids in psychological conditions, as most of these studies used relatively small sample sizes and failed to control for additional medications and long-term adverse effects (Sachs et al., 2015).

There is good evidence that exogenous cannabinoids can lower intraocular pressure in individuals with glaucoma. However, to have a clinically significant impact, the dose and frequency of use need to be extremely high, which can increase the likelihood of negative side effects (Sachs et al., 2015).

In conclusion, although there is evidence of the therapeutic potential of cannabinoids in the treatment of various clinical conditions, there are still serious concerns regarding potential deleterious side effects. A large systematic review on adverse reactions associated with the use of cannabinoids for therapeutic purposes demonstrated that their short-term use leads to an increased risk of non-serious adverse events, including mild to moderate sedation, dizziness, dry mouth, nausea and lack of attention (Wang et al., 2008). Therefore, further studies are needed to better understand the long-term effects of cannabinoid use for various therapeutic indications.

## 4 Final comments and future perspectives

“Cannabis” is the most popular drug in the Western world and, despite the potential harms associated with its use, the prevalence of cannabinoid-based drug use has become increasingly popular in recent decades, leading to extensive research focused on the safety, toxicology, and potency of this group of substances, as well as their therapeutic potential.

Despite the level of uncertainty that still exists regarding the safety and efficacy of medicinal and recreational use of cannabis, which continues to fuel intense debate about the best policies to implement globally and in each country in particular, there are some topics on which the existing information is more consistent. Indeed, it is recognized that consumption causes unequivocal acute cardiovascular, respiratory, cognitive, psychological and general public health effects, and persistent cardiovascular and respiratory consequences in chronic users are well documented. Evidence on other long-term impacts of “cannabis” is mixed and probably influenced by age at first use, duration and frequency of use, as well as potency and pre-existing comorbidities.

Currently, there is still no global harmonization on how the therapeutic use of cannabinoids should be legislated, designed and implemented, leading to some inconsistency regarding several key aspects, namely: i) standards for access and use of these compounds by patients; ii) the rights of caregivers; iii) the role of mandatory medical prescriptions; iv) product safety and packaging requirements, among other potentially relevant ones. In this context of lack of harmonized rules, it is up to each country to make its own decisions and regulate in the way it sees fit.

Indeed, the medicinal properties of Cannabis and its components have been the subject of intense scientific research for decades, in an attempt to circumvent the various controversies and respond to the great commercial interest. It is, however, very important to highlight the difference between these medicines, composed of purified preparations, in relation to the consumption of the plant. These preparations eliminate variability, both in their composition and also in terms of administration routes.

Undeniably, it has been demonstrated that there is a wide variation in the content and potency of cannabinoids, in the mode of administration and in the form of cannabinoid consumed, leading to a change in their bioavailability and, consequently, in the response to their use. This is particularly important when comparing the consumption of cannabinoids for recreational use and their use for therapeutic purposes. Collectively, these factors contribute to the great difficulty in deciphering the relative safety and efficacy of cannabinoids, both in the medical and recreational contexts. On the other hand, the literature on medicinal and recreational “cannabis” suggests clear discordance between current state policies, public opinion and scientific evidence, the latter often inconclusive and others biased by methodological inconsistencies, making it difficult to obtain highly robust evidence and delaying the implementation of appropriate precautions.

Thus, in the absence of further clinical trials in humans to support their use, and with current scientific evidence, cannabinoids are still considered products with potential therapeutic benefit, as an alternative or adjuvant to the pharmacological options currently indicated for various clinical situations, but are not, however, the first choice. Some areas that require more evidence and greater consistency are long-term safety, possible adverse effects on the health of vulnerable people and those with pre-existing pathologies, as well as precise information on the guidelines to be followed. In fact, it would be very important for both doctors, who will be responsible for eventually recommending the use of cannabinoids for medicinal purposes, and patients potentially chosen for their use, to benefit from more robust information and a more clarifying discussion about their potential risks and benefits.

Finally, it should be noted that there is recent evidence of the adverse effects of synthetic cannabinoids which, although similar to those of phytocannabinoids, are apparently more potent and long-lasting. It is therefore with great concern that in Portugal, as in other European countries, there has been a constant and rapid emergence of NPS, a rate that exceeds the means for any legislative control. The degree of physical and psychological dependence caused by these substances is similar to that caused by many illicit substances, and may even exceed it in certain situations. Furthermore, in some cases, a causal link has been clinically identified between consumption and the development of psychiatric disorders, including psychotic episodes, neurological disorders and serious cardiac complications. Furthermore, substances whose effects on human physiology are often still poorly understood circulate on this market, which makes it very difficult to treat acute poisoning and identify and treat long-term effects.

It is concluded that the issue of recreational consumption of cannabinoids or their use for therapeutic purposes is a topic of great medical and scientific interest, particularly when we intend to weigh up the pros and cons for human health. Considering the global trend to reduce or even eliminate the prohibition of this type of compound, it is crucial to establish constant intersections between public health policies and advances in scientific knowledge. In fact, we are witnessing extensive research into the safety, toxicology, potency and therapeutic potential of cannabinoids in various indications, the results of which should constitute the pillar for the implementation of policies, both at the level of potential use for therapeutic purposes and recreational consumption.
